# Dietary vasodilator and vitamin C/L-arginine/choline blend improve broiler feed efficiency during finishing and reduce woody breast severity at 6 and 7 wks

**DOI:** 10.1016/j.psj.2022.102421

**Published:** 2022-12-10

**Authors:** M.M. Meyer, E.A. Bobeck

**Affiliations:** Department of Animal Science, Iowa State University, Ames, IA 50011, USA

**Keywords:** broiler, woody breast, antioxidant, vasodilator, amino acid

## Abstract

Woody breast has become a considerable economic concern to the poultry industry. This myopathy presents rigid, pale breasts characterized by replacement of lean muscle protein with connective tissue, a result of hypoxia and oxidative stress in a metabolically starved muscle with inadequate circulation. Hence, the objectives were to supplement broiler diets with ingredients specifically aimed to improve circulation and oxidative status. About 1,344 male Ross 708 broilers were assigned to 1 of 4 diets: 1) a basal diet (**control**), 2) basal diet plus a blend of 0.2% supplemental L-arginine, 0.17% choline bitartrate, and 0.03% vitamin C (**blend**), 3) 0.1% vasodilator ingredient (**vasodilator**), or 4) 0.02% Astaxanthin ingredient (**AsX**). At d 14, 28, 42, and 49, performance outcomes were collected on all birds and serum from 16 broilers/diet (*n* = 64) was analyzed for creatine kinase and myoglobin. Once weekly beginning on d 28, a subset of 192 broilers were measured for breast width. On d 42 and 49, breast fillets from 16 broilers/diet (*n* = 64) were palpated for woody breast severity, weighed, and analyzed for compression force at 1-day postmortem and water-holding capacity at 2-day postmortem. mRNA was isolated from 15 breast fillets/timepoint for qPCR quantification of myogenic gene expression. Data were analyzed using Proc Mixed (SAS Version 9.4) with the fixed effect of diet. Feed conversion ratio was improved in the blend and vasodilator-fed birds d 42 to 49, each by over 2 points (*P* < 0.05). Breast width was increased in the control on d 42 compared to the vasodilator and AsX-fed broilers (*P* < 0.05). At d 42, there were 12% greater normal fillets in blend diet-fed birds and 13% more normal scores in vasodilator-fed birds at d 49 compared to the control. At d 49, myogenin expression was upregulated in the AsX diet compared to blend and control diets (*P* < 0.05), and muscle regulatory factor-4 expression was increased by 6.5% in the vasodilator diet compared to the blend and AsX diets (*P* < 0.05). Blend and vasodilator diets simultaneously improved feed efficiency in birds approaching market weight while reducing woody breast severity.

## INTRODUCTION

A muscle disorder known as woody breast (**WB**) has come to the forefront of meat quality concerns in the U.S. broiler industry in the last 10 yr (Baldi et al., 2020). WB was first described in the literature in 2014 as abnormally hard and pale breast tissue with histological abnormalities including myodegeneration and significant fibrosis (Sihvo et al., 2014). Estimated WB prevalence in 2016 was 5 to 10% of chicken breasts produced in the United States, an issue costing the industry over $200 million annually (Kuttappan et al., 2016). The cost of WB is likely even greater today; current data show an industry value increased by $4 billion and a production increase of approximately 5 billion pounds since 2016 (USDA-NASS, 2022). The etiology of WB remains unknown, but occurrence and severity consistently increase with broiler age and weight (Kuttappan et al., 2017a). This disorder is also widely associated with heavier, thicker breast fillets (Mallman et al., 2019; Hasegawa et al., 2020; Zhang et al., 2021).

Multiple meat quality issues including reduced water-holding capacity (Tijare et al., 2016), diminished meat color (Kuttappan et al., 2017b), increased compression force (Mudalal et al., 2014; Sun et al., 2018a), and increased shear force are observed in WB tissue compared to normal breast muscle (Trocino et al., 2015; Bowker and Zhuang, 2019). A characteristic rigidity/loss of breast tenderness is evident at harvest and is the hallmark trait of WB, hence a subjective, tactile scoring system with varying degrees ranging from normal to severe WB has been widely adopted for identification and grading (Tijare et al., 2016; Kuttappan et al., 2017a). Histologically, insufficient blood supply due to reduced capillary to muscle fiber ratio observed in WB muscles results in metabolically starved tissue and degeneration (Velleman, 2019). An altered metabolic state has been described through proteomic analysis demonstrating reduced carbohydrate metabolism and hindered energy production in the primarily white fast-twice glycolytic breast muscle fibers of broilers (Kuttappan et al., 2017a). Localized hypoxia from lack of circulation is the cause of resulting muscle atrophy (Maharjan et al., 2020). Hypoxia and oxidative stress in affected muscle is evidenced through shifted plasma metabolites and altered gene expression in WB muscle (Mutryn et al., 2015; Greene et al., 2019; Lilburn et al., 2019; Papah and Abasht, 2019).

Serum creatine kinase has been measured in relation to broiler exercise; Mattioli et al. (2017) showed that exercise increased serum creatine kinase activity, identifying this skeletal muscle-specific protein as a potential biomarker of muscle-derived metabolic stress when present in circulation. Further, Santos et al. (2021) showed that plasma creatine kinase as well as WB incidence were increased in conventional fast-growing broilers compared to slower-growing strains. Myoglobin presence in the serum is indicative of muscle damage (Brancaccio et al., 2010), but has not yet been associated with WB. However, an increase in breast muscle myoglobin gene expression has been reported by Mutryn et al. (2015), making this protein relevant in terms of WB.

While it has been acknowledged that a reduction in broiler body weight or breast proportions would be successful in reducing WB occurrence (Lilburn et al., 2019), dietary interventions have improved meat quality outcomes and metabolic state without sacrificing breast yields. Estevez and Petracci (2019) supplemented dietary magnesium, an antioxidant, and saw increased plasma catalase activity and reduced WB incidence as a result. Multiple dietary treatments including supplemental arginine, vitamin C, and vitamin premix, as well as reduced amino acid density during the grower phase were tested by Bodle et al. (2018), and the authors saw reduced WB severity. L-arginine acts as a vasodilator through nitric oxide production, hence serves to improve blood flow and oxygen supply, likely preventing the hypoxic state characteristic to WB. Vitamin C acts as an antioxidant by scavenging free radicals, therefore supplementation may have reduced WB by mediating oxidative stress. Reduced amino acid availability during the grower phase was also effective in reducing WB; Bodle et al. (2018) hypothesized that this allowed breast muscle recovery and satellite cell development following the intense starter phase of growth.

A study feeding increased arginine:lysine ratios saw reduced WB severity (Zampiga et al., 2019), and a recent study aimed to improve antioxidant status through nutrition and saw improvement in WB scores when feeding Ethoxyquin in combination with mineral methionine hydroxy analog chelates of zinc, copper, manganese, and organic selenium (Kuttappan et al., 2021). Awadh and Zangana (2021) fed broilers Astaxanthin, an antioxidant carotenoid, and saw improved carcass quality characteristics including meat water-holding capacity and color. Alternatively, Greene et al. (2019) supplemented broiler diets with quantum blue, a phytase, and saw 5% reduced WB compared to the control diet. The authors concluded that this was an effect of improved oxygen homeostasis, evidenced by gene expression.

Inositol-stabilized arginine silicate (**ASI**) has not yet been tested as a broiler supplement to mitigate WB but has been proven to increase blood arginine levels and nitric oxide in humans, effectively driving vasodilation and increasing blood flow during exercise (Rood-Ojalvo et al., 2015). This ability makes ASI a potential vasodilator supplement in broiler diets. Supplemental dietary choline has recently been explored in terms of broiler meat quality; work by Gregg et al. (2022) showed that additional choline did not affect WB scores, however, abdominal fat pad was reduced in broilers raised to 32 d. Further changes in carcass quality may have been observed at a greater age of bird. The role of choline in lipid metabolism makes it relevant concerning WB, as affected tissue is widely reported to show increased adipose infiltration (Lilburn et al., 2019; Mallman et al., 2019). Therefore, the current study objectives were to supplement broiler diets with ingredients specifically aimed to improve circulation and oxidative status, some novel and some previously shown to be beneficial, with the hypothesis that they would reduce WB severity.

## MATERIALS AND METHODS

### Animals

All live animal work was approved by the Iowa State University Institutional Animal Care and Use Committee, Protocol #19-156. About 1,344 male Ross 708 broilers were transported from Welp Hatchery (Bancroft, IA) to 2 identical rooms in the Iowa State University Robert T. Hamilton Poultry Research and Teaching Farm (Ames, IA) on day of hatch. Chicks were weighed as pens of 21 upon arrival (mean BW = 43.6 ± 0.11 g) and raised for 49 d. Feed and water were provided ad libitum from hanging feeders and nipple waterlines, and basal diets were formulated according to Ross 708 Nutrition Specifications for 3.10 to 3.50 kg live weight (Aviagen, 2019). Birds were inspected 3 times daily and high/low temperatures and humidity were recorded daily from June to July 2021. Temperatures were gradually reduced over time, starting at approximately 90°F d 0 to 14, averaging 80°F d 14 to 28, and finishing at approximately 70°F d 28 to 49. Temperatures were reduced to 75°F over d 34 to 35 and to approximately 70°F for the remainder of the study. Birds were gradually adjusted from 24 h light (100% daylight) on d 0 to 20 h light: 4 h dark by d 8 (20–25 lux) for the remainder of grow-out. The birds experienced heat stress from d 32 to 34, where temperatures averaged 92°F across both rooms. This heat stress was not a part of the study design but anticipated as likely to occur due to birds being raised during the summer.

### Diets

Birds were randomly assigned to diet on d 0, with 16 pens/diet. The 4 diets in this study, with ingredient sourcing and specifications, are described in detail in Table 1. Dietary treatments included a basal diet (**control**), basal diet plus 0.2% L-arginine, 0.03% vitamin C, and 0.17% choline bitartrate (**blend**), basal diet plus 0.1% ASI ingredient (**vasodilator**), and basal diet plus 0.02% Astaxanthin ingredient (**AsX**). Supplementation levels of L-arginine, vitamin C, choline bitartrate, and Astaxanthin used were based off of existing broiler work using the ingredients, but as the vasodilator ingredient has not been previously fed to broilers, 0.1% was used in the current work as a starting point. Basal diet formulation and proximate analyses of each diet by performance phase are provided in Table 2.

**Table 1:** Dietary treatments fed to male Ross 708 broilers.

Control	Basal diet
Blend	Basal diet + 0.2022% L-arginine^1^ + 0.0301% vitamin C^2^ + 0.1737% choline bitartrate^3^
Vasodilator	Basal diet + 0.10% Inositol-stabilized arginine silicate^4^
AsX	Basal diet + 0.02% Carophyll Pink^5^ (Astaxanthin)

1 L-arginine, 99.6%; Shine Star Biological Engineering Co., Ltd., Gong An, Jingzhou, Hubei, China.

2 Vitamin C; Shandong Luwei Pharmaceutical Co., Ltd., Zibo City, Shandong, China.

3 Choline bitartrate, 98%, Petshure, New Hampton, NY.

4 Proprietary Inositol-stabilized arginine silicate ingredient.

5 Carophyll Pink; 10% Astaxanthin, DSM Nutritional Products Europe Ltd., Basel, Switzerland.

**Table 2:** (A–D) Diet formulations and proximate analyses fed to Ross 708 broilers over 49 d in (A) Starter, d 0 to 14, (B) Grower, d 14 to 28, (C) Finisher 1 (d 28–42), and Finisher 2 (d 42–49).

A				
Ingredients	Basal starter diet^1^			
Corn	54.89%			
Soybean meal 48	37.30%			
Soy oil	2.20%			
Salt	0.40%			
DL methionine	0.36%			
Lysine HCl	0.25%			
Threonine	0.10%			
Limestone	1.05%			
Dicalcium phosphate	2.01%			
Choline chloride 60	0.41%			
Vitamin premix^2^	0.63%			
Calculated values				
Fat (%)	4.74			
Crude protein (%)	23.07			
ME (kcal/kg)	3004.42			
Digestible lysine (%)	1.31			
Digestible arginine (%)	1.39			
Digestible threonine (%)	0.88			


Analyzed values (dry matter)	Control	Blend	Vasodilator	AsX
Dry matter (%)	88.40	88.74	88.86	88.87
Crude fat (%)	5.80	5.47	5.34	5.98
Crude protein (%)	21.76	22.50	22.78	22.12
Gross energy (kcal/kg)	4248.50	4268.40	4206.92	4169.78

B				
Ingredients	Grower basal diet^1^			
Corn	58.21%			
Soybean meal 48	33.60%			
Soy oil	3.15%			
Salt	0.36%			
DL Methionine	0.30%			
Lysine HCl	0.20%			
Threonine	0.05%			
Limestone	0.90%			
Dicalcium phosphate	1.81%			
Choline chloride 60	0.38%			
Vitamin premix^2^	0.63%			
Calculated values				
Fat (%)	5.73			
Crude protein (%)	21.46			
ME (kcal/kg)	3100			
Digestible lysine (%)	1.18			
Digestible arginine (%)	1.28			
Digestible threonine (%)	0.78			

Analyzed values (dry matter)	Control	Blend	Vasodilator	AsX
Dry matter (%)	88.60	88.31	88.45	88.66
Crude fat (%)	6.57	6.48	6.21	6.60
Crude protein (%)	20.45	20.83	20.40	20.82
Gross energy (kcal/kg)	4289.34	4335.47	4307.83	4304.90

C				
Ingredients	Finisher 1 basal diet^1^			
Corn	62.00%			
Soybean meal 48	29.20%			
Soy oil	4.15%			
Salt	0.36%			
DL methionine	0.26%			
Lysine HCl	0.16%			
Threonine	0.03%			
Limestone	0.83%			
Dicalcium phosphate	1.60%			
Choline chloride 60	0.37%			
Vitamin premix^2^	0.63%			
Calculated values				
Fat (%)	6.79			
Crude protein (%)	19.60			
ME (kcal/kg)	3200			
Digestible lysine (%)	1.04			
Digestible arginine (%)	1.16			
Digestible threonine (%)	0.70			

Analyzed values (dry matter)	Control	Blend	Vasodilator	AsX
Dry matter (%)	89.75	90.16	89.81	89.23
Crude fat (%)	7.81	6.94	7.56	7.56
Crude protein (%)	19.16	19.65	18.28	18.19
Gross energy (kcal/kg)	4377.17	4436.65	4375.71	4351.83

D				
Ingredients	Finisher 2 basal diet^1^			
Corn	66.32%			
Soybean meal 48	25.00%			
Soy oil	3.98%			
Salt	0.40%			
DL methionine	0.25%			
Lysine HCl	0.22%			
Threonine	0.05%			
Limestone	0.80%			
Dicalcium phosphate	1.52%			
Choline chloride 60	0.42%			
Vitamin premix^2^	0.63%			
Calculated values				
Fat (%)	6.75			
Crude protein (%)	17.99			
ME (kcal/kg)	3224.03			
Digestible lysine (%)	0.97			
Digestible arginine (%)	1.04			
Digestible threonine (%)	0.65			

Analyzed values (dry matter)	Control	Blend	Vasodilator	AsX
Dry matter (%)	89.12	89.37	89.28	89.12
Crude fat (%)	7.11	7.74	7.09	6.90
Crude protein (%)	17.65	17.50	17.83	17.48
Gross energy (kcal/kg)	4328.78	4375.03	4331.53	4333.52

1 The basal diet was formulated with 0.406% filler: control was completed with 0.406% sand; blend with 0.2022% L-arginine + 0.0301% vitamin C + 0.1737% choline bitartrate; vasodilator with 0.1000% Inositol-stabilized arginine silicate + 0.306% sand; AsX with 0.0200% Carophyll Pink 10 + 0.386% sand.

2 Vitamin and mineral premix provided per kg of diet: selenium 250 μg; vitamin A (retinyl acetate) 8,250 IU; cholecalciferol (vitamin D_3_) 2,750 IU; α-tocopherol acetate (vitamin E) 17.9 IU; menadione 1.1 mg; vitamin B_12_ 12 μg; biotin 41 μg; choline 447 mg; folic acid 1.4 mg; niacin 41.3 mg; pantothenic acid 11 mg; pyridoxine 1.1 mg; riboflavin 5.5 mg; thiamine 1.4 mg; iron 282 mg; magnesium 125 mg; manganese 275 mg; zinc 275 mg; copper 27.5 mg; iodine 844 μg.

### Performance

The 7-wk trial was separated into Starter (wk 0–2), Grower (wk 2–4), Finisher 1 (wk 4–6), and Finisher 2 (wk 6–7) performance periods. All birds were weighed as a pen at the conclusion of each period, and mean body weight and body weight gain were calculated and averaged by bird. Feed intake was recorded by measuring disappearance throughout. Feed conversion ratio was calculated by pen and averaged per bird.

### Breast Yield and Meat Quality

On d 28, 35, 42, and 49, a subset of 48 wing-banded birds/diet were measured for breast width. The width of the entire pectoralis major muscle from beneath wings was measured using a soft seamstress tape measure. One randomly selected broiler/pen was sampled at d 42 and 49 for breast muscle collection. Following euthanasia by carbon dioxide (AVMA, 2020) the entire left breast fillet was dissected from 64 birds/timepoint and weighed individually (*n* = 128 breasts). Following dissection, WB score was assigned on the same fillets by one researcher using a tactile 0 to 3 scale based on the scoring system described by Kuttappan et al. (2016). Breasts were scored on a 3-point scale where normal represented a fillet that retained flexibility throughout, moderate WB indicated a fillet with hardness through cranial and potentially caudal region but retained flexibility in the medial region, and severe WB represented a fillet with no flexibility.

Left fillets were placed in approximately 39°F cold storage overnight and compression force was measured 1-day postmortem using methods described in Sun et al. (2018a). Briefly, each raw fillet was compressed 3 times on different points across the cranial region with a 6.5 mm flat probe on a TA.XT Plus Texture Analyzer (Texture Technologies Corp., Surrey, UK). A trigger force of 5 g was used, probe height was set at 55 mm, pre-and postprobe speeds used were 10 mm/s, and test speed was 5 mm/s. The mean compression force of the 3 tests per fillet was used for data analysis. Following texture analysis, fillets were returned to cold storage overnight, and 2-day postmortem, the same left fillets were analyzed for water-holding capacity. A centrifugation method was used, where exactly 10 g of minced tissue in duplicate were centrifuged at 25,000 × *g* for 20 min, the supernatant was removed, and the weight of the remaining solid was recorded. Values are reported as a percent centrifugation loss, indicating a negative relationship with water-holding capacity of the meat, and were calculated as follows:

%Centrifugationloss=[(initialweight−post−spinweight)/(initialweight)]×100

### Breast Muscle mRNA Isolation and qPCR

On d 42 and 49, approximately 52 cm^2^ samples from the cranial and medial regions from a subset of right breast fillets were collected (*n* = 30 samples, 15 fillets/timepoint). Samples were flash frozen in liquid nitrogen and stored at −80°C for analysis. RNA was isolated from fifteen breast muscle samples/diet across both timepoints using the RNeasy Fibrous Tissue Kit (Qiagen, Germantown, MD). Isolated RNA was DNAse treated and diluted to 50 ng/µL for One-Step SYBR Green quantitative PCR (Qiagen, Germantown, MD), then amplified using 6 muscle-growth and regulation genes of interest and normalized with the ubiquitously expressed chicken housekeeping gene, 28s. Primers included: myoblast determination protein 1 (**MyoD**); myogenin (**MyoG**); muscle regulatory factor-4 (**MRF4**); insulin-like growth factor 1 (**IGF1**); insulin-like growth factor 2 (**IGF2**); and myostatin (**MSTN**). Means are reported as adjusted Ct values, calculated as follows:

$$40-[(\text{MeanC}t_{\text{test}\;\text{gene}}+\left[\left(\text{Media}n_{28s}\right)-\left(\text{MeanC}t_{28S}\right)\right]$$

### Serum Creatine Kinase and Myoglobin

Blood was collected from one randomly selected broiler/pen (*n* = 16 birds/diet) at d 14, 28, 42, and 49 into serum separation tubes (BD Vacutainer, BD Biosciences, Franklin Lakes, NJ) and centrifuged at 1,000 × *g* for 15 min. Serum was collected and split for a creatine kinase assay and a myoglobin ELISA. The creatine kinase analysis was conducted at the Iowa State University Clinical Pathology Laboratory (Ames, IA) using dry chemistry analysis for abundance. The myoglobin assay was conducted with serum diluted 1:4 in PBS following manufacturer protocols and the Chicken Myoglobin Competitive ELISA (MyBioSource, San Diego, CA).

### Statistical Analysis

All data were analyzed using SAS software version 9.4 (SAS Institute Inc., Cary, NC). PROC UNIVARIATE was used to assess the data distribution prior to analysis. WB categorical data were analyzed as percent distribution of scores by diet using a chi-square likelihood ratio. The section of breast sampled for qPCR (cranial vs. medial) did not affect expression of any genes tested (*P* > 0.05), hence data from both sections were pooled for remaining data analysis. The performance, breast muscle yield, meat quality, qPCR, and serum data were normally distributed and were analyzed using PROC MIXED, a mixed linear model, with the main effect of diet. A post hoc Tukey-Kramer adjustment was used for all pairwise comparisons between diets, and pairwise differences between diets are reported with or without a significant main effect of diet from ANOVA. A value of *P* ≤ 0.05 was considered significant and *P* ≤ 0.10 is reported as a trend.

## RESULTS

### Performance

Broiler feed intake was impacted by diet in the Starter period; birds fed AsX ate 26 g more than birds fed the blend diet and 16 g more than the control (*P* = 0.006). In the Finisher 1 period, pairwise comparisons showed that birds fed the AsX diet ate 60 g more than those fed the blend diet (*P* = 0.169; Table 3). Body weight gain trended to be affected by diet in the Starter period (*P* = 0.108); in a pairwise comparison of diets, vasodilator-fed birds gained more than those fed AsX during this period (Table 3). There was a trend for an effect of diet on body weight at d 14 (*P* = 0.099), with vasodilator-fed birds weighing 10 g more than AsX-fed broilers (Table 3). At d 28, a pairwise comparison between diets showed that birds fed the vasodilator weighed 20 g more than AsX-fed birds (*P* = 0.193). Feed conversion ratio was increased in the AsX diet during the Starter period compared to all other diets (*P* = 0.002). During the Finisher 1 period, diet trended to impact FCR (*P* = 0.069), with blend diet fed-birds showing an improved FCR compared to the vasodilator and AsX diets. Blend and vasodilator diets improved FCR during the Finisher 2 period (*P* = 0.042), with reductions by over 2 FCR points in both diets was observed.

**Table 3:** Ross 708 male broiler performance outcomes averaged per bird by the main effect of diet^1^.

Performance outcome (bird avg)	Control	Blend	Vasodilator	AsX	SEM	*P* value
Feed intake, kg						
Starter (g)	519^bc^	509^c^	529^ab^	535^a^	5.30	0.006
Grower	1.34	1.34	1.33	1.32	0.01	0.555
Finisher 1	2.05^ab^	2.01^b^	2.03^ab^	2.07^a^	0.02	0.169
Finisher 2	1.19	1.18	1.21	1.20	0.03	0.895
						
Body weight gain, kg						
Starter (g)	363^ab^	367^ab^	369^a^	359^b^	3.10	0.108
Grower	0.90	0.90	0.91	0.90	0.01	0.690
Finisher 1	1.17	1.18	1.15	1.18	0.02	0.295
Finisher 2	0.56	0.59	0.59	0.55	0.03	0.665
						
Body weight, kg						
d 14 (g)	407^ab^	411^ab^	412^a^	402^b^	3.20	0.099
d 28	1.31^ab^	1.31^ab^	1.32^a^	1.30^b^	0.01	0.193
d 42	2.47	2.50	2.47	2.48	0.02	0.489
d 49	3.00	3.07	3.05	3.03	0.03	0.350
						
FCR						
Starter	1.43^b^	1.39^b^	1.43^b^	1.49^a^	0.02	0.002
Grower	1.49	1.48	1.47	1.48	0.01	0.543
Finisher 1	1.75^ab^	1.70^b^	1.77^a^	1.75^a^	0.02	0.069
Finisher 2	2.30^a^	2.05^b^	2.08^b^	2.21^ab^	0.07	0.042

1 ^abc^Different superscript letters indicate post hoc pairwise differences between diets within the same timepoint.

### Breast Yield and Meat Quality

**Figure 1:**
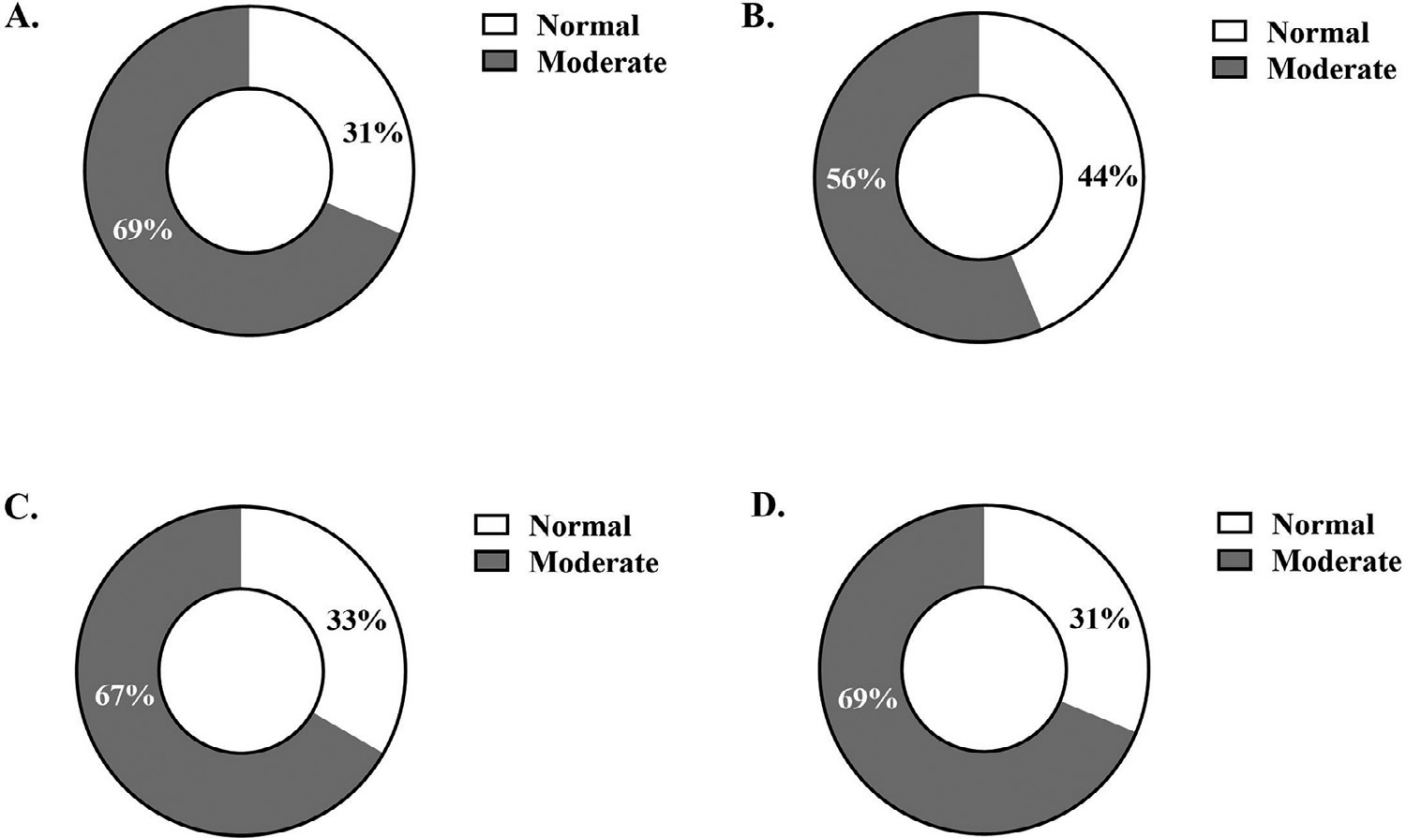
Ross 708 broiler woody breast palpation scores conducted on left breast fillets at d 42 harvest. Scores are presented as percent distribution by diet: (A) control, (B) blend, (C) vasodilator, and (D) AsX.

**Figure 2:**
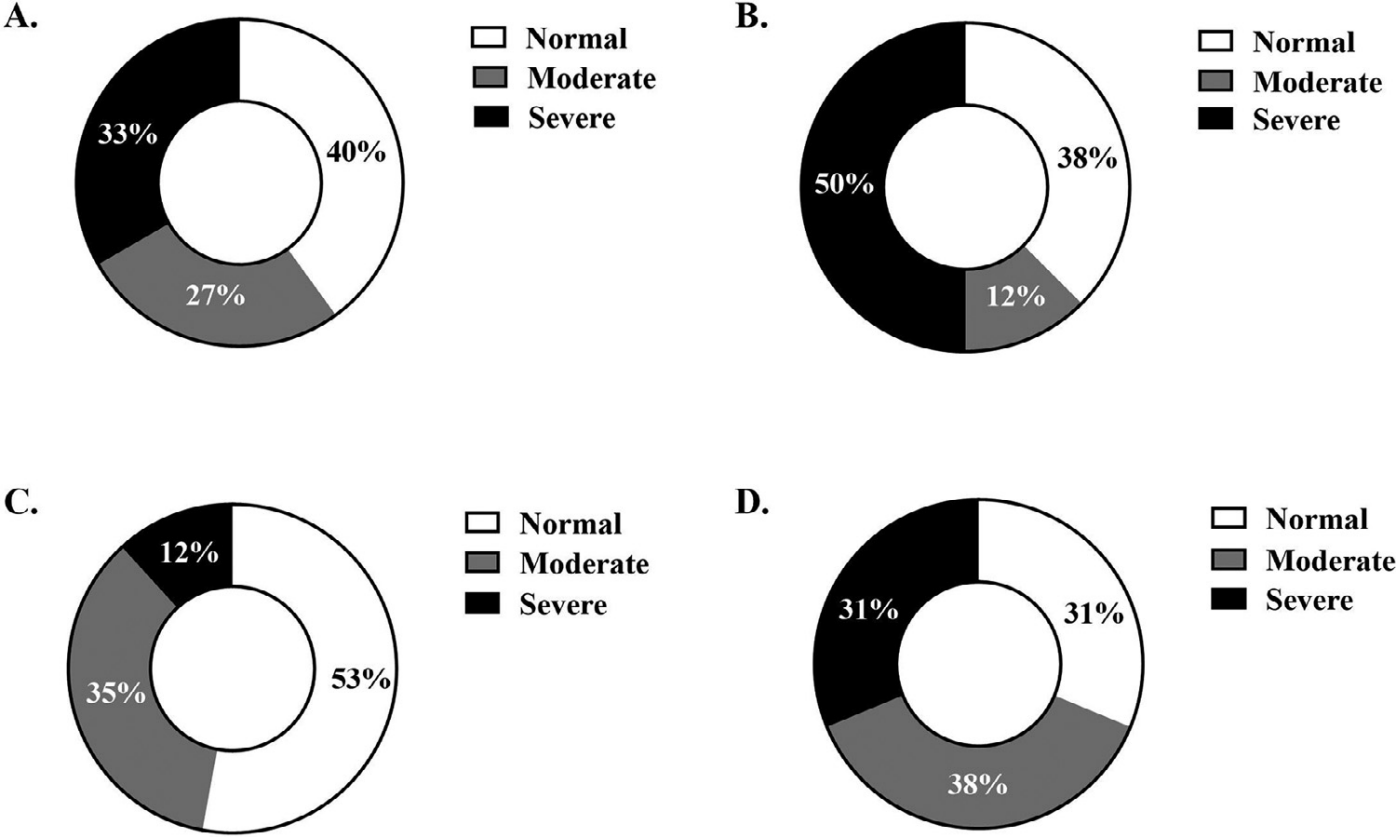
Ross 708 broiler woody breast palpation scores conducted on left breast fillets at d 49 harvest. Scores are presented as percent distribution by diet: (A) control, (B) blend, (C) vasodilator, and (D) AsX.

Breast width was affected by diet at d 42 (*P* = 0.020), with control birds showing wider breasts than vasodilator or AsX-fed birds (Table 4). Breast fillet weight was not affected by diet at d 42 or 49. At d 42, WB scores in all diets ranged from normal-moderate, with the greatest percentage of normal fillet scores observed in the blend diet. This amounted to a 12.5% increase in unaffected fillets and a 12.5% reduction in severe WB scores compared to the control (*P* = 0.646; Figure 1). At d 49, WB scores ranged from normal to severe, with the greatest percentage of normal breasts observed in the vasodilator diet. 13% more vasodilator-fed broiler breasts were scored normal and 22% less fillets were scored severe compared to the control (*P* = 0.310; Figure 2). Meat quality measures taken 1- and 2-day postmortem, compression force and water-holding capacity, were unaffected by diet at d 42 or 49 sampling (Table 4).

**Table 4:** Breast yield and meat quality outcomes measured on live birds: breast width, and on the left breast fillet postmortem: breast weight, compression force, and water-holding capacity^1^. Data were analyzed with the main effect of diet^2^.

Breast outcome	Control	Blend	Vasodilator	AsX	SEM	*P* value
Live bird measure						
Breast width, cm						
d 28	16.73	16.82	16.76	16.45	0.14	0.220
d 35	19.33	19.08	19.05	19.08	0.15	0.495
d 42	21.71^a^	21.34^ab^	21.15^b^	21.01^b^	0.17	0.020
d 49	22.67	22.88	22.43	22.43	0.24	0.445
Breast fillet measure						
Breast fillet weight, kg						
d 42	0.32	0.29	0.31	0.30	0.01	0.425
d 49	0.41	0.41	0.40	0.39	0.01	0.758
Compression force, g (1-day postmortem)						
d 42	2199.4	2126.2	2113.5	1946.7	101.1	0.293
d 49	2430.6	2574.5	2489.1	2577.4	108.1	0.699
Centrifugation loss^1^, % (2-day postmortem)						
d 42	6.03	7.25	6.19	7.40	0.91	0.616
d 49	3.29	4.44	4.37	4.12	0.56	0.447

1 Negative relationship with water-holding capacity.

2 ^ab^Different superscript letters indicate post hoc pairwise differences between diets within the same timepoint.

### Breast Muscle Gene Expression

**Figure 3:**
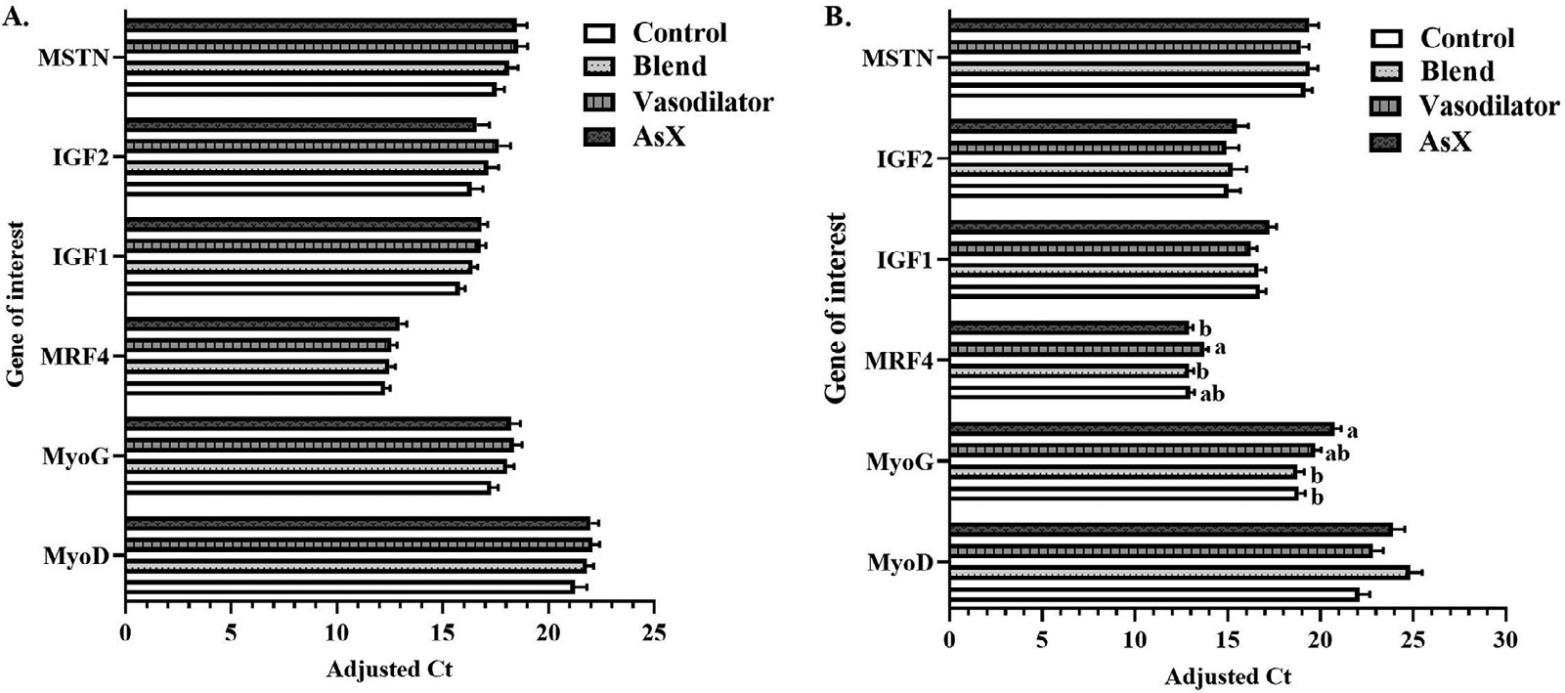
Adjusted cycle threshold (Ct) values obtained from one-step real-time qPCR using RNA extracted from breast tissue at (A) d 42 and (B) d 49 using 15 breast fillets/timepoint and 2 segments from each breast/timepoint. Data presented are LSMeans (±SEM) by day, analyzed with the main effect of diet.

No changes in myogenic gene expression were found at d 42 (Figure 3A); however, at d 49, changes in amplified genes were detected (Figure 3B). MyoG expression was affected by diet (*P* = 0.001), with an upregulation of 10.8% in the AsX diet compared to the blend diet and 10.3% compared to the control. MRF4 expression was increased in the vasodilator diet compared to the blend and AsX diets by 6.5% (*P* = 0.019), while expression was not different from the control (Figure 3B). MyoD expression trended to be upregulated in the blend diet (*P* = 0.088), with an Adjusted Ct increase of 12.4% compared to the control. Remaining genes amplified were not affected by diet.

### Serum Creatine Kinase and Myoglobin

Serum creatine kinase was not altered by the main effect of diet at any timepoint, but pairwise differences existed between diets at d 14 and 42. Creatine kinase presence was increased in blend diet-fed birds compared to vasodilator fed birds by 63% at d 14, while neither differed from the control (*P* = 0.129; Table 5). At d 42, control-fed birds showed 72% greater serum creatine kinase than AsX-fed broilers (*P* = 0.118; Table 5), while blend and vasodilator-fed birds were intermediate. Serum myoglobin was not altered by diet (Table 5).

**Table 5:** Ross 708 broiler serum creatine kinase and myoglobin from 1 bird/pen (*n* = 64) on d 14, 28, 42, and 49 analyzed with the main effect of diet^1^.

Serum measure	Control	Blend	Vasodilator	AsX	SEM	*P* value
Serum CK (iu/L)						
d 14	2,726^b^	3,198^a^	1,961^b^	3,038^ab^	400.7	0.129
d 28	7,978	8,790	7,025	6,511	1,029.5	0.368
d 42	31,869	27,646	37,450	36,961	4,975.8	0.443
d 49	49,022^a^	45,634^ab^	41,793^ab^	28,565^b^	6,450.6	0.118
Serum myoglobin (ng/uL)						
d 14	181.35	177.34	170.32	173.44	5.93	0.5728
d 28	165.37	165.09	169.68	167.07	4.81	0.8738
d 42	201.95	205.33	203.92	206.12	4.97	0.9218
d 49	204.98	199.66	203.33	203.5	2.71	0.4472

1 ^ab^Different superscript letters indicate post hoc pairwise differences between diets within the same timepoint.

## DISCUSSION

In light of the rising need to maximize sustainable broiler production, nutritional approaches that can maintain pounds of meat produced while improving protein quality are highly relevant. It has been suggested that WB may be an attempt by the muscle itself to adapt to the proportion of growth stimulated by genetic selection, and that any further selection pressure on breast yields will be limited by the muscle's biological potential and animal welfare (Baldi et al., 2020). Multiple novel dietary approaches were tested in the current study, including a vitamin C/L-arginine/choline blend, a vasodilator supplement, and a carotenoid antioxidant. Distribution of WB scores did not differ statistically in the current study, but practically meaningful differences existed between diets.

In 6-wk-old broilers, the blend diet showed an advantage compared to the control, with 44% of fillets scoring normal and 56% showing moderate WB. This outcome agrees with previous work by Bodle et al. (2018), who fed supplemental arginine and vitamin C and saw reduced WB scores in each diet. In contrast to their findings, we did not observe reduced breast size due to vitamin C inclusion, as breast width nor fillet weight were different from the control at 42 d. Work characterizing the metabolic state in WB muscle tissue by Abasht et al. (2016) suggested a possible overactivation of the vitamin C-synthesis pathway causing depletion of glycogen and therefore oxidative stress, leading Bodle et al. (2018) to summarize that vitamin C supplementation likely improved oxidative state and reduced muscle damage through alleviating metabolic demands from this pathway. In addition to the free-radical scavenging ability that makes vitamin C an effective antioxidant (Traber and Stevens, 2011), previous work in broilers has shown that supplemental arginine in combination with vitamin C improved cardiopulmonary vascular performance; the authors concluded this to be a synergistic effect of increased nitric oxide availability and reduced oxidative stress (Bautista-Ortega and Ruiz-Feria, 2010).

On the other hand, improvements in WB scores due to the blend diet at wk 6 in the current study may relate to the heat-stress event where temperatures reached over 32°C during a 2-day period. An increased percentage of normal breast fillets following a mild heat stress challenge was similarly observed by Kuttappan et al. (2021) when supplementing a mineral methionine hydroxy analog chelate of zinc, copper, and manganese in combination with an antioxidant and organic selenium. It has been well-documented that during times of external stress such as heat, housing, or feed-restriction, endogenous vitamin C synthesis may be impaired and vitamin C can be depleted, hence supplementing vitamin C early in life has been shown to improve body weight and FCR in poultry (Lopes et al., 2020). In the case of heat stress, specifically, an animal welfare concern that reduces feed intake and weight gain in broilers (Van Hieu et al., 2022), vitamin C supplementation decreases corticosteroid synthesis and improves weight gain, FCR, and carcass quality (Sahin et al., 2003; Kumar et al., 2014). Therefore, it is possible that the blend diet was particularly advantageous during the heat stress challenge due to vitamin C, a conclusion supported by the performance data. Blend diet-fed birds showed the numerically highest body weights at d 42 and improved feed conversion compared to the vasodilator and AsX diets during the Finisher 1 period, as well as significantly improved feed conversion compared to the control at d 49.

Choline is well-known to prevent fatty liver in poultry and plays a key role in methylation of homocysteine to form methionine, an essential amino acid to protein-building and the first rate-limiting amino acid in conventional poultry diets (Zeisel and Blusztajn, 1994). Deficient methionine contributes to oxidative stress (Caballero et al., 2010), and work in laying hens by Dong et al. (2019) showed increased egg yolk lipids and reduced liver fat content with choline supplementation. The authors concluded that supplemental choline may have increased methionine synthesis and improved oxidative state. In addition to a possible benefit to oxidative status, supplementing choline to broilers has been shown to reduce abdominal fat pad (Gregg et al., 2022). However, choline bitartrate has not been previously studied as a dietary WB-mediator. Determining potential changes in lipid metabolism with supplemental choline in relation to the reduced WB severity observed in the current study warrants future research, that is, measuring fat content in breast tissue. Notably, in the molecular analysis, there was a trend for increased MyoD expression in breasts from broilers fed the blend diet compared to the control (*P* < 0.10). MyoD is skeletal muscle-specific marker of muscle cell differentiation (Velleman et al., 2014), and has been previously linked with muscle fiber growth vs. fat deposition in broiler breast tissue, providing some evidence for muscle vs. increased fat content in blend-fed broiler fillets (Sachs et al., 2019). Further, L-arginine in the blend diet may have improved WB severity at d 42 through increased nitric oxide production driving vasodilation, an effect evidenced in broilers by Bautista-Ortega and Ruiz Feria (2010) and observed through WB reduction by Bodle et al. (2018).

Increased blood flow could be paramount to preventing the characteristic hypoxia and metabolically starved WB phenotype. Nitric oxide is a strong vasodilator made from L-arginine (Bowen et al., 2007), and reduced nitric oxide has been associated with pulmonary hypertension in broilers (Tan et al., 2007). A dietary L-arginine increase of 30% by Zampiga et al. (2019) showed a similar reduction in WB severity to the current work, providing support for this amino acid as a WB mediator. Inositol-stabilized ASI, the vasodilator ingredient in the current study, has not been previously fed to broilers. This form of arginine has shown improved bioavailability and absorption compared to L-arginine in rats and humans (Proctor et al., 2007; Komorowski and Ovaljo, 2016), and proven boosted nitric oxide levels in humans (Gills et al., 2021). As a human workout supplement, the ASI ingredient used has been shown to significantly increase circulating arginine and nitric oxide (Rood-Ojalvo et al., 2015).

Our data provide evidence for a similar effect in broilers. Birds fed the vasodilator diet had 53% normal WB scores at d 49 and only 12% of fillets scored severe. This is in comparison to 33% severe scores in the control diet at this timepoint. This positive outcome at 7-wk of age is accompanied by a performance benefit through improved feed efficiency in the Finisher 2 phase (vasodilator FCR of 2.08 vs. 2.30 in the control; *P* < 0.05). Notable in the current work is that supplemental L-arginine inclusion in the blend diet (0.2%) vs. ASI inclusion in the vasodilator diet (0.1%) yielded similar results in WB improvement, perhaps highlighting the improved bioavailability of arginine from the ASI ingredient at half the inclusion rate. Further, while breast width was reduced by 0.56 cm at d 42 in the vasodilator diet compared to the control, this may have been an effect of healthy muscle growth and regulation as demonstrated by increased MRF4 expression at d 49 in vasodilator-fed broiler breast tissue compared to the blend or AsX diets. MRF4 expression is related to myoblast proliferation and hypertrophy, and a lack of this protein causes myopathy (Velleman et al., 2014). Further, MRF4 muscle expression has been shown to be upregulated with exercise in pigs and broilers, highlighting its role as a negative regulator in normal muscle growth (Yin et al., 2015; Kalbe et al., 2018). Providing more evidence that healthy muscle development was stimulated due to vasodilator inclusion is the reduction of serum creatine kinase observed at d 14; creatine kinase is a skeletal muscle-specific protein indicating muscle damage when in circulation (Brancaccio et al., 2010), and plasma creatine kinase has been previously linked to WB (Santos et al., 2021). Due to the novelty of this ingredient and the promising meat quality and molecular results reported here, future research supplementing broiler diets with ASI to improve blood flow to the breast muscle is warranted.

Astaxanthin is a microalgae-derived carotenoid that acts as a potent antioxidant and induces multiple meat quality benefits when fed to broilers, including improved meat color (Akiba et al., 2001; Sun et al., 2018b), water-holding capacity and pH (Awadh and Zangana, 2021), and texture (Perenlei et al., 2014; Pertiwi et al., 2022). Astaxanthin-supplemented broiler diets result in improved tissue antioxidant status and increased expression of genes related to lipid metabolism, redox status, and stress response in the livers of broilers exposed to heat stress (Sun et al., 2018b; Tolba et al., 2020). In contrast to improved meat texture reported by Perenlei et al. (2014), we did not observe improvements in WB scores nor compression force, a mechanical measure of meat texture, due to AsX. Breast muscle width was reduced in this diet compared to the control at 6-wk, body weight was reduced at d 14 and 28 compared to the vasodilator diet, and FCR was worse compared to the control during the Starter period and compared to the blend-fed birds during the Finisher 1 period. This outcome may be unsurprising, as Astaxanthin has previously been associated with unimproved performance in broilers (Perenlei et al., 2014), and has been linked to worse feed conversion in birds finished under high ambient temperatures (Sun et al., 2018b), but is in contrast to work showing improved weight gain and feed conversion during the finisher period (Jeong and Kim, 2014). Results observed in heat stressed broilers by Sun et al. agree with the reduced feed efficiency we observed during the heat stress-containing Finisher 1 period and may indicate increased metabolic demands associated with absorbing Astaxanthin and depositing pigment from the carotenoid into muscle.

However, Sun et al. (2018b) also reported improved tissue antioxidant capacity in broilers fed Astaxanthin that were reared in temperatures 10°F higher than commercial recommendations from wk 4 to 6, and Tolba et al. (2020) saw upregulated hepatic gene expression related to redox status, inflammation, and lipid metabolism in Astaxanthin-fed broilers exposed to heat stress, indicating a positive relationship between oxidative status and Astaxanthin in high temperature conditions. In the current study, there were improvements in measures of muscle damage and myogenic growth in the AsX diet. Serum creatine kinase was reduced in broilers aged 49 d compared to the control and reduced numerically compared to all other diets, indicating a protective effect. MyoG expression was upregulated in AsX-fed broiler breast tissue compared to the control and blend diets at the same time-point. MyoG is a muscle regulatory factor necessary for myoblast differentiation and ultimate formation of myotubes and adult muscle fibers (Velleman et al., 2014). Expression in the breast muscle has been increased with broiler exercise (Yin et al., 2015), and decreased with feed restriction during the first week of life (Velleman et al., 2014). Considered with the existing literature on feeding Astaxanthin, our data warrant further research into this ingredient, including ideal applications for dietary intervention, although do not currently support it as an effective tool against WB.

Dietary mitigations to WB remain of interest to broiler producers and researchers, and the work described here presents viable options: a vitamin C, choline bitartrate, and L-arginine blend, as well as an ASI ingredient. Both diets show the ability to reduce WB occurrence and severity in 6- or 7-wk broiler carcasses without decreasing body weight or breast yields, and improved feed conversion during wk 6 to 7 of life. Further research is warranted optimizing these ingredients for WB prevention, and future work examining the Astaxanthin ingredient under different environmental conditions is of interest.
